# Supplementary material for: A unified neurocognitive model of semantics language social behaviour and face recognition in semantic dementia
Source: Nat Commun. 2020 May 22;11:2595. doi: 10.1038/s41467-020-16089-9 (PMC7244491; doi:10.1038/s41467-020-16089-9)
Supplement: Supplementary file 1 — Supplementary Information [file 41467_2020_16089_MOESM1_ESM.pdf]

Supplementary Information

A unified neurocognitive model of semantics language social behaviour and face recognition in semantic dementia

Junhua DING *et al.*

Supplementary Table 1. The significant clusters of the two-tailed univariate VBCM analysis

	Peak coordinate	Peak R value	Voxel number	Side	Regions
Factor 1: apathy	-4.5 61.5 4.5	0.40	141	L	Superior frontal gyrus, medial part
	-6 48 22.5	0.36	140	L	Superior frontal gyrus, medial part
	3 61.5 -12	0.38	111	R	Superior frontal gyrus, medial orbital part
	-4.5 61.5 -12	0.37	73	L	Superior frontal gyrus, medial orbital part
Factor 2: face	55.5 -28.5 -6	0.76	13791	R	Insula; fusiform, parahippocampal , rectus, anterior cingulate and Heschl gyri; superior, middle and inferior temporal gyri; temporal pole; inferior frontal gyrus, orbital part; olfactory cortex; superior frontal gyrus, medial orbital; Rolandic operculum
	-1.5 31.5 -16.5	0.60	474	L	Rectus and anterior cingulate gyri; superior frontal gyrus, medial orbital part
	4.5 -3 34.5	0.60	411	R	Anterior and middle cingulate gyri
	55.5 -27 21	0.54	82	R	Rolandic operculum
	-1.5 -3 37.5	0.51	78	L	Middle cingulate gyrus
Factor 3: naming	-37.5 -16.5 -37.5	0.71	4557	L	Superior, middle and inferior temporal gyri; temporal pole
	-21 3 -25.5	0.59	797	L	Temporal pole: superior temporal gyrus; parahippocampal gyrus
Factor 4: disinhibition	51 6 -12	0.32	83	R	Temporal pole: superior temporal gyrus
	3 13.5 -24	0.35	76	R	Rectus gyrus
	58.5 3 -1.5	0.35	52	R	Temporal pole: superior temporal gyrus
Factor 5: SD severity	-33 18 -10.5	0.52	3736	L	Insula; rectus, superior temporal and Heschl gyri; superior and inferior frontal gyrus: orbital part;
	-1.5 -15 42	0.52	2543	L	Anterior and middle cingulate gyri; supplementary motor area; superior frontal gyrus: medial and medial orbital parts; olfactory cortex; Rolandic operculum
	1.5 -4.5 48	0.53	2179	R	Anterior and middle cingulate gyri; supplementary motor area; superior frontal gyrus: medial and medial orbital parts;
	-67.5 -33 -4.5	0.46	1921	L	Middle and inferior temporal gyri; inferior occipital gyrus
	-49.5 -48 45	0.51	665	L	Inferior parietal and supramarginal gyri
	4.5 27 -28.5	0.39	579	R	Superior frontal gyrus, orbital
	-16.5 -22.5 -25.5	0.42	454	L	Parahippocampal gyrus
	34.5 24 -1.5	0.38	297	R	Insula
	-49.5 -61.5 -22.5	0.37	175	L	Inferior temporal gyrus
	60 -52.5 15	0.35	155	R	Middle temporal gyrus
	48 -49.5 46.5	0.45	108	R	Inferior parietal gyrus
	64.5 -15 12	0.41	77	R	Superior temporal gyrus
	-60 -6 4.5	0.36	72	L	Superior temporal gyrus
	18 -19.5 -27	0.35	53	R	Parahippocampal gyrus
	-52.5 -45 9	0.39	52	L	Middle temporal gyrus

Only positive clusters were reported (Cluster size > 50 voxels; n = 47; df = 45). The results of F2 & 3 were corrected by the permutation-based FWER (v =100). Their *q* values are 0.02 and 0.007, respectively. Other results' thresholds are uncorrected $p < 0.05$. VBCM: voxel-based correlation mapping. Source data are provided as a Source Data file.

Supplementary Table 2. The significant clusters of the two-tailed SVR analysis

	Peak coordinate	Peak R value	Voxel number	Side	Regions
Factor 1: apathy	-55.5 3 -7.5	8.73	1750	L	Temporal pole; inferior frontal gyrus: orbital part; middle temporal gyrus
	-4.5 60 -13.5	9.22	1373	L	Superior frontal gyrus: medial and medial orbital parts; anterior cingulate and rectus gyri
	4.5 60 -16.5	9.02	879	R	Superior frontal gyrus: medial and medial orbital parts; anterior cingulate and rectus gyri
	-60 -43.5 34.5	7.16	175	L	Supramarginal gyrus
	-31.5 -24 9	5.16	150	L	Insula
	-51 -63 39	9.27	120	L	Angular gyrus
	4.5 40.5 43.5	5.57	90	R	Superior frontal gyrus: medial part
	-3 -4.5 43.5	4.35	78	L	Middle cingulate gyrus
	-51 24 1.5	8.27	68	L	Inferior frontal gyrus: orbital part
Factor 2: face	22.5 6 -36	10	4127	R	Middle and superior temporal gyri; insula; parahippocampal and fusiform gyri; temporal pole; Rolandic operculum
	4.5 10.5 -9	7.96	373	R	Olfactory cortex; superior frontal gyrus: medial orbital part
Factor 3: naming	-55.5 4.5 -9	10	9025	L	Superior, middle and inferior temporal gyri; temporal pole; fusiform and parahippocampal gyri; insula
	-27 -43.5 -10.5	6.23	170	L	Fusiform gyrus
	-57 -58.5 -13.5	5.99	155	L	Inferior temporal gyrus
	-61.5 -54 4.5	7.06	94	L	Middle temporal gyrus
	-64.5 -22.5 1.5	4.09	56	L	Middle temporal gyrus
Factor 4: disinhibition	54 9 -10.5	10	2502	R	Temporal pole; insula; superior and middle temporal gyri
	6 10.5 -13.5	9.82	877	R	Olfactory cortex; rectus gyrus; temporal pole
	69 -31.5 3	7.59	119	R	Middle temporal gyrus
	27 -4.5 -39	5.95	100	R	Fusiform gyrus
	16.5 -4.5 -24	4.71	77	R	Parahippocampal gyrus
	-16.5 12 -24	4.57	63	L	Inferior frontal gyrus: orbital part
	-57 -67.5 3	4.73	63	L	Middle temporal gyrus
	55.5 -12 -7.5	4.35	52	R	Superior temporal gyrus
Factor 5: SD severity	-7.5 31.5 -25.5	10	6914	L	Insula; anterior and middle cingulate gyri; rectus and Heschl gyri; superior frontal gyrus: medial orbital and orbital parts; olfactory cortex; supplementary motor area; inferior frontal gyrus: orbital part; Rolandic operculum; caudate
	-64.5 -43.5 -1.5	9.50	2686	L	Middle and inferior temporal gyri; inferior occipital gyrus
	4.5 -3 45	7.19	2463	R	Anterior and middle cingulate gyri; supplementary motor area; superior frontal gyrus: medial orbital and medial parts
	9 25.5 -25.5	7.92	864	R	Superior and inferior frontal gyri: orbital part
	-49.5 -51 46.5	9.92	767	L	Inferior parietal and angular gyri
	-13.5 -42 0	7.08	585	L	Parahippocampal and fusiform gyri
	34.5 22.5 -1.5	9.92	439	R	Insula
	60 -54 12	6.40	244	R	Middle temporal gyrus
	48 -49.5 48	7.43	120	R	Inferior parietal gyrus
	21 -21 -25.5	4.23	110	R	Parahippocampal gyrus
	64.5 -15 10.5	5.62	95	R	Superior temporal gyrus
	66 -36 7.5	6.04	87	R	Middle temporal gyrus
	-58.5 3 1.5	4.66	68	L	Superior temporal gyrus
	-55.5 -43.5 9	5.55	54	L	Middle temporal gyrus

Only positive clusters were reported (Cluster size > 50 voxels; n = 47). The cross-validation *p* values of F1-F5 were 0.18, 0.17, 0.002, 0.04, 0.17, respectively. SVR: support vector regression. Source data are provided as a Source Data file.

Supplementary Table 3. The significant clusters of the two-tailed SCCAN

	Peak Coordinate	Peak R value	Voxel number	Side	Regions
Factor 1: apathy	-31.5 18 -25.5	0.82	3324	L	Temporal pole; superior and middle temporal gyri; inferior frontal gyrus: orbital part
	4.5 48 9	0.48	753	R	Anterior cingulate gyrus; superior frontal gyrus: medial orbital and medial parts
	-52.5 -60 36	0.48	599	L	Angular, supramarginal and inferior parietal gyri
	-3 48 -9	0.22	400	L	Superior frontal gyrus: medial orbital part; anterior cingulate gyrus
	-4.5 46.5 19.5	0.36	348	L	Superior frontal gyrus: medial part; anterior cingulate gyrus
	-42 1.5 7.5	0.27	288	L	Insula; Rolandic operculum
	-33 -22.5 13.5	0.26	160	L	Insula
	54 -3 3	0.20	122	R	Superior temporal gyrus
	39 24 -18	0.18	90	R	Inferior frontal gyrus: orbital part
	48 13.5 -37.5	0.15	85	R	Temporal pole: middle temporal gyrus
	-52.5 -37.5 -1.5	0.21	79	L	Middle temporal gyrus
	-58.5 -34.5 -18	0.16	76	L	Inferior temporal gyrus
	-55.5 -46.5 6	0.18	76	L	Middle temporal gyrus
Factor 2: face	31.5 -15 -31.5	1	1813	R	Fusiform and parahippocampal gyri; temporal pole: middle temporal gyrus
	54 -28.5 -4.5	0.89	1022	R	Superior and middle temporal gyri
	37.5 -7.5 15	0.68	696	R	Insula; Rolandic operculum
	3 33 -15	0.64	473	R	Olfactory cortex; superior frontal gyrus: medial orbital part; anterior cingulate gyrus
	4.5 1.5 31.5	0.82	443	R	Anterior and middle cingulate gyri
	-1.5 33 -15	0.40	265	L	Rectus and anterior cingulate gyri; superior frontal gyrus: medial orbital part
	-24 9 -16.5	0.55	237	L	Insula
	-3 1.5 37.5	0.49	107	L	Middle cingulate gyrus
	40.5 -48 -24	0.44	83	R	Fusiform gyrus
Factor 3: naming	-37.5 -15 -37.5	1	5989	L	Superior, middle and inferior temporal gyri; temporal pole; parahippocampal and fusiform gyri; insula
	-57 -63 -9	0.27	134	L	Inferior temporal gyrus
	-27 -45 -7.5	0.23	61	L	Fusiform gyrus
Factor 4: disinhibition	-57 -66 3	0.56	310	L	Middle temporal gyrus
	-33 19.5 -24	0.40	298	L	Temporal pole: superior temporal gyrus; inferior frontal gyrus: orbital part
	-19.5 -18 -27	0.55	202	L	Parahippocampal gyrus
	4.5 15 -19.5	0.25	120	R	Rectus gyrus
	-34.5 16.5 -7.5	0.25	115	L	Insula
	58.5 3 -3	0.32	106	R	Temporal pole: superior temporal gyrus
	58.5 -63 10.5	0.21	55	R	Middle temporal gyrus
	36 16.5 -6	0.20	54	R	Insula
Factor 5: SD severity	-40.5 6 -3	0.87	5062	L	Insula; superior temporal, rectus and Heschl gyri; superior and inferior frontal gyri: orbital part; Rolandic operculum; olfactory cortex; temporal pole: superior temporal gyrus; caudate
	-63 -43.5 -1.5	0.79	3367	L	Middle and inferior temporal gyri; inferior occipital gyrus
	3 -1.5 43.5	0.89	2243	R	Anterior and middle cingulate gyri; supplementary motor area; superior frontal gyrus: medial part
	-3 -16.5 43.5	0.69	1764	L	Anterior and middle cingulate gyri; superior frontal gyrus: medial part; supplementary motor area
	-18 -22.5 -24	0.53	1182	L	Parahippocampal and fusiform gyri; temporal pole: middle temporal gyrus
	13.5 28.5 -25.5	0.59	858	R	Superior and inferior frontal gyri: orbital part
	-51 -54 45	1	677	L	Inferior parietal, angular and supramarginal gyri
	36 24 -3	0.42	416	R	Insula
	-27 -46.5 -4.5	0.63	185	L	Lingual gyrus
	60 -52.5 13.5	0.43	171	R	Middle temporal gyrus
	18 -27 -19.5	0.39	158	R	Parahippocampal gyrus
	49.5 -49.5 48	0.65	145	R	Inferior parietal gyrus
	-61.5 -37.5 18	0.44	133	L	Superior temporal gyrus
	64.5 -15 10.5	0.63	108	R	Superior temporal gyrus
	28.5 9 -18	0.23	86	R	Insula
	3 48 -4.5	0.29	79	R	Superior frontal gyrus: medial orbital part
	-37.5 -30 -15	0.28	62	L	Fusiform gyrus
	-1.5 49.5 -4.5	0.25	58	L	Superior frontal gyrus: medial orbital part

Only positive clusters were reported (Cluster size > 50 voxels; n = 47). The cross-validation *p* values of F1-F5 were 0.08, 0.00003, 0.00002, 0.17, 0.04, respectively. SCCAN: sparse canonical correlation analysis for neuroimaging. Source data are provided as a Source Data file.

Supplementary table 4. The R values of right face-related ROIs in SD patients.

	OFA	FFA	pSTS	vATL	aSTS
Face					
Word-picture verification	0.27 (-0.02-0.52)$^{0.07}$	0.43 (0.15-0.64)$^{0.003}$	0.30 (0.01-0.54)$^{0.04}$	0.46 (0.20-0.66)$^{0.001}$	0.55 (0.31-0.73)$^{0.00007}$
Picture matching	0.27 (-0.02-0.51)$^{0.07}$	0.36 (0.08-0.58)$^{0.01}$	0.27 (-0.02-0.52)$^{0.06}$	0.42 (0.15-0.63)$^{0.004}$	0.39 (0.12-0.61)$^{0.006}$
Picture naming	0.12 (-0.17-0.40)$^{0.41}$	0.09 (-0.20-0.37)$^{0.54}$	0.00 (-0.28-0.30)$^{0.98}$	-0.01 (-0.30-0.28)$^{0.93}$	0.05 (-0.24-0.33)$^{0.76}$
Perception	0.38 (0.09-0.60)$^{0.01}$	0.25 (-0.06-0.51)$^{0.11}$	0.37 (0.08-0.60)$^{0.01}$	0.39 (0.10-0.61) $^{0.01}$	0.42 (0.14-0.64)$^{0.005}$
Object					
Word-picture verification	0.07 (-0.22-0.35)$^{0.64}$	0.07 (-0.23-0.35)$^{0.65}$	-0.02 (-0.31-0.27)$^{0.87}$	0.04 (-0.25-0.33)$^{0.77}$	0.19 (-0.11-0.45)$^{0.21}$
Picture matching	0.01 (-0.28-0.29)$^{0.96}$	0.23 (-0.07-0.48)$^{0.13}$	0.05 (-0.24-0.33)$^{0.76}$	0.38 (0.10-0.60)$^{0.009}$	0.35 (0.08-0.58)$^{0.01}$
Picture naming	-0.07 (-0.35-0.22)$^{0.65}$	-0.02 (-0.30-0.27)$^{0.91}$	-0.13 (-0.40-0.16)$^{0.37}$	-0.16 (-0.43-0.13)$^{0.28}$	-0.07 (-0.35-0.22)$^{0.62}$
Perception	0.16 (-0.15-0.43)$^{0.31}$	-0.13 (-0.41-0.17)$^{0.40}$	0.09 (-0.21-0.38)$^{0.57}$	-0.24 (-0.50-0.06)$^{0.12}$	-0.19 (-0.46-0.11)$^{0.21}$

Two-tailed Pearson correlations were used (word-picture verification: n = 46, df = 44; perception: n = 44, df = 42; others: n = 47; df = 45). The numbers in parentheses are the 95% confidence intervals. The numbers in superscripts are the uncorrected P values. OFA: occipital face area; FFA: fusiform face area; pSTS: posterior superior temporal sulcus; aSTS: anterior superior temporal sulcus; ATL: anterior temporal lobe. Source data are provided as a Source Data file.

Supplementary table 5. The R values of left face-related ROIs in SD patients.

	OFA	FFA	pSTS	vATL	aSTS
Face					
Word picture verification	0.14 (-0.16-0.41)$^{0.37}$	0.18 (-0.11-0.45)$^{0.22}$	0.25 (-0.04-0.50)$^{0.09}$	0.35 (0.07-0.58)$^{0.02}$	0.22 (-0.08-0.48)$^{0.14}$
Picture matching	0.09 (-0.20-0.37)$^{0.55}$	0.33 (0.05-0.57)$^{0.02}$	0.35 (0.07-0.58)$^{0.02}$	0.43 (0.16-0.64)$^{0.003}$	0.34 (0.06-0.57)$^{0.02}$
Picture naming	0.04 (-0.25-0.33)$^{0.77}$	0.25 (-0.04-0.50)$^{0.08}$	0.13 (-0.17-0.40)$^{0.40}$	0.29 (0.01-0.54)$^{0.05}$	0.33 (0.05-0.56)$^{0.02}$
Perception	0.23 (-0.07-0.50)$^{0.13}$	0.09 (-0.21-0.38)$^{0.55}$	0.19 (-0.11-0.46)$^{0.21}$	0.30 (0.00-0.55)$^{0.05}$	0.03 (-0.27-0.32)$^{0.85}$
Object					
Word picture verification	0.03 (-0.26-0.32)$^{0.82}$	0.30 (0.01-0.54)$^{0.04}$	0.19 (-0.10-0.46)$^{0.20}$	0.44 (0.17-0.64)$^{0.003}$	0.43 (0.16-0.64)$^{0.003}$
Picture matching	-0.01 (-0.30-0.28)$^{0.95}$	0.19 (-0.11-0.45)$^{0.21}$	0.10 (-0.20-0.37)$^{0.52}$	0.43 (0.16-0.64)$^{0.003}$	0.17 (-0.12-0.43)$^{0.26}$
Picture naming	0.01 (-0.28-0.29)$^{0.96}$	0.47 (0.21-0.67)$^{0.0008}$	0.21 (-0.08-0.47)$^{0.15}$	0.52 (0.27-0.70)$^{0.0002}$	0.52 (0.28-0.70)$^{0.0002}$
Perception	0.08 (-0.22-0.37)$^{0.59}$	0.13 (-0.17-0.41)$^{0.39}$	0.18 (-0.12-0.45)$^{0.24}$	0.13 (-0.17-0.41)$^{0.40}$	0.11 (-0.19-0.39)$^{0.47}$

Two-tailed Pearson correlations were used (word-picture verification: n = 46, df = 44; perception: n = 44, df = 42; others: n = 47; df = 45). The numbers in parentheses are the 95% confidence intervals. The numbers in superscripts are the uncorrected P values. OFA: occipital face area; FFA: fusiform face area; pSTS: posterior superior temporal sulcus; aSTS: anterior superior temporal sulcus; ATL: anterior temporal lobe. Source data are provided as a Source Data file.

Supplementary table 6. The R values of left and right face related ROIs in normal controls.

	OFA	FFA	pSTS	vATL	aSTS
Face & right ROIs					
Word picture verification	-0.19 (-0.58-0.28)$^{0.44}$	-0.30 (-0.65-0.17)$^{0.21}$	0.27 (-0.19-0.64)$^{0.24}$	-0.12 (-0.54-0.34)$^{0.61}$	-0.23 (-0.61-0.24)$^{0.33}$
Picture matching	0.13 (-0.33-0.54)$^{0.59}$	-0.42 (-0.73-0.03)$^{0.07}$	-0.18 (-0.58-0.29)$^{0.45}$	-0.46 (-0.75- -0.02)$^{0.04}$	-0.32 (-0.67-0.14)$^{0.17}$
Picture naming	0.04 (-0.41-0.47)$^{0.85}$	-0.29 (-0.65-0.17)$^{0.21}$	0.33 (-0.13-0.68)$^{0.15}$	-0.04 (-0.47-0.41$^{0.88}$	-0.18 (-0.58-0.28)$^{0.44}$
Perception	0.14 (-0.33-0.55)$^{0.57}$	-0.14 (-0.55-0.32)$^{0.58}$	0.20 (-0.27-0.59)$^{0.40}$	0.08 (-0.38-0.50)$^{0.74}$	0.06 (-0.40-0.49)$^{0.80}$
Face & left ROIs					
Word picture verification	0.06 (-0.39-0.49)$^{0.80}$	0.08 (-0.38-0.50)$^{0.75}$	-0.30 (-0.66-0.16)$^{0.20}$	-0.33 (-0.67-0.14)$^{0.16}$	-0.07 (-0.50-0.38)$^{0.77}$
Picture matching	-0.03 (-0.47-0.42)$^{0.90}$	-0.39 (-0.71-0.07)$^{0.09}$	0.27 (-0.20-0.64)$^{0.25}$	-0.54 (-0.79- -0.13)$^{0.01}$	-0.13 (-0.54-0.34)$^{0.60}$
Picture naming	0.13 (-0.33-0.54)$^{0.59}$	-0.02 (-0.46-0.42)$^{0.92}$	-0.07 (-0.50-0.39)$^{0.77}$	-0.20 (-0.59-0.27)$^{0.41}$	-0.13 (-0.54-0.33)$^{0.58}$
Perception	0.28 (-0.19-0.64)$^{0.23}$	-0.26 (-0.63-0.21)$^{0.28}$	0.03 (-0.42-0.47)$^{0.91}$	-0.02 (-0.46-0.42)$^{0.93}$	0.02 (-0.43-0.46)$^{0.91}$

Two-tailed Pearson correlations were used (n = 20, df = 18). The numbers in parentheses are the 95% confidence intervals. The numbers in superscripts are the uncorrected P values. OFA: occipital face area; FFA: fusiform face area; pSTS: posterior superior temporal sulcus; aSTS: anterior superior temporal sulcus; ATL: anterior temporal lobe. Source data are provided as a Source Data file.
